# Prescription patterns for children with juvenile idiopathic arthritis in Michigan Medicaid: a comparison by prescriber type

**DOI:** 10.1186/1546-0096-12-38

**Published:** 2014-09-05

**Authors:** Meredith P Riebschleger, Heather A Van Mater, Lisa M Cohn, Sarah J Clark

**Affiliations:** Division of Pediatric Rheumatology, Child Health Evaluation and Research Unit, University of Michigan, 6C15A NIB, 300 North Ingalls, Ann Arbor, MI 48109 USA; Division of Pediatric Rheumatology, Duke University, Durham, NC USA

**Keywords:** Arthritis, Juvenile rheumatoid, Physician’s practice patterns, Drug therapy

## Abstract

**Background:**

Due to a limited number and disparate distribution of pediatric rheumatologists in the US, a variety of physician types provide care to children with rheumatologic diseases. However, little is known about how that care may differ across prescribing physician groups. Our objective was to compare medication claims for children with juvenile idiopathic arthritis (JIA) by type of prescribing physician.

**Methods:**

We performed a retrospective cohort study of children with JIA using Michigan Medicaid data for 7/1/2005-6/30/2007, employing descriptive and bivariate analyses by age, medication type, and prescriber type.

**Results:**

Among 397 children, there was no difference in the frequency of medication claims for children with internist versus pediatric rheumatologist prescribers. Children with non-rheumatologist prescribers were less likely to have claims for disease modifying anti-rheumatic drugs (DMARDs) and biologic agents.

**Conclusion:**

Differential use of DMARDs and biologic agents by rheumatologists indicates the importance of referring children with JIA for specialty care.

## Background

Previous studies have indicated that in many areas of the United States, children do not have easy access to a pediatric rheumatologist, due to both the small absolute number of pediatric rheumatologists and their concentration in academic centers [1–3]. Internist rheumatologists – who are more numerous and more geographically dispersed than their pediatric colleagues – may therefore play a prominent role in the care of children with rheumatologic conditions [4–6]. Some children may receive care from primary care physicians or non-rheumatology specialists for their rheumatologic disease [7]. The variety of physician types providing care to children with rheumatologic diseases is of special concern to primary care physicians, who must determine when and where to refer their patients with rheumatologic complaints. Concerns about specialist availability are particularly salient in states such as Michigan, which has large rural areas, and numerous medically underserved areas (MUAs) and health professional shortage areas (HPSAs) in both urban and rural settings.

Juvenile idiopathic arthritis (JIA) is the most common of the pediatric rheumatologic conditions, with a prevalence of approximately 60 cases per 100,000 children [8]. Studies have indicated that time-to-treatment with DMARDs or biologic agents is an important factor in response to these drugs for children with JIA [9, 10]. Although several studies have addressed the participation of internist rheumatologists and primary care physicians in the care of children with JIA [4–7], it is unclear whether children with JIA receive different medications, depending upon their prescribing physician type. As such, primary care physicians, policy makers, and parents are missing a critical piece of information as they decide where to send children with possible JIA. The goal of this study was to explore prescription patterns for medications commonly used to treat JIA based upon prescriber type.

## Methods

This study was approved by the University of Michigan Medical School Institutional Review Board. Administrative claims data were obtained from Michigan Medicaid for 7/1/2005-6/30/2007. The study population was limited to children 21 years of age or younger, with Medicaid enrollment for ≥11 months in at least one study year, and with no other insurance coverage. A sensitivity analysis including only those children 15 years of age or younger was also performed.

To minimize misclassification, children were defined as having JIA if they had at least 1 claim for a medication commonly used to treat JIA, and at least 1 visit coded for a JIA diagnosis (ICD-9-CM 714.30, 714.31, 714.32, 714.33, 714.0, 696.0, 720.0, 720.89). Lab and radiology tests were not considered visits. Children with ICD-9-CM codes for other rheumatic diseases (710.xx) were excluded, as those diseases may include arthritis but would supersede a diagnosis of JIA.

Demographic information included age and race. Pharmacy claims included National Drug Codes and prescriber identification numbers. Medications commonly used to treat JIA included non-steroidal anti-inflammatory drugs (NSAIDs), disease modifying anti-rheumatic drugs (DMARDs), biologic agents, and any other medication prescribed by a rheumatologist. Using prescriber identification numbers linked to Medicaid provider specialty data, prescribers were classified as pediatric rheumatologists, internist rheumatologists, non-rheumatology specialists (which included all physicians who were neither rheumatologists nor primary care physicians), primary care physicians (which included general pediatricians, family practitioners, and general internists), or hospital/unknown. To verify the accuracy of this classification, one author (HvM) reviewed the list of prescribing physicians by hand. Children with multiple prescriber types were placed in the group of the most specialized prescriber, as that physician was presumed to be directing the overall care. Children whose prescribers were only in the hospital/unknown group were excluded from analyses investigating prescribing patterns.

Descriptive analyses included counts and proportions. Chi square tests were used for bivariate analyses. P values <0.05 were considered statistically significant. Analyses were performed using STATA version 10.

## Results

**Figure 1:**
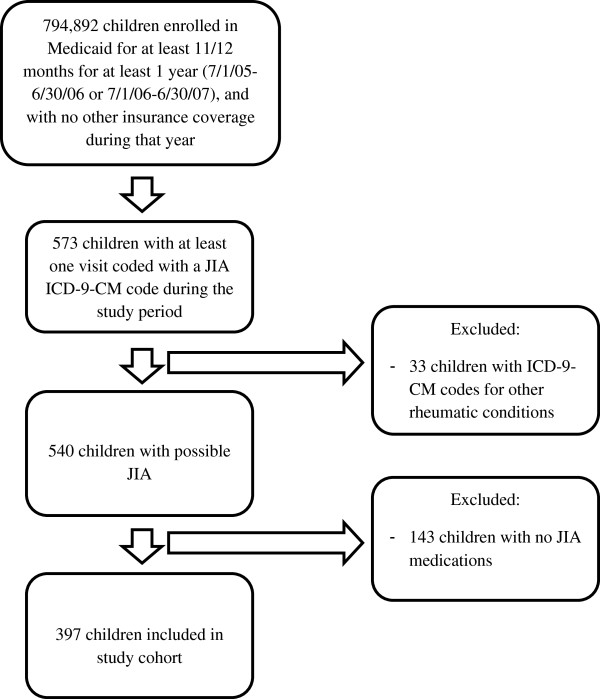
**Cohort diagram.**

Figure 1 shows the application of eligibility criteria, which yielded a study population of 397 children. Of those, 61% were Caucasian and 30% were African American; 12% were 0–5 years of age, 19% were 6–11 years of age, 36% were 11–15 years of age, and 34% were 16–21 years of age.

By definition, all children in the cohort had a medication claim, but 84 children (21%) had prescriptions only from prescribers in the hospital/unknown group. Among the remaining 313 children, 188 (47% of the cohort) had at least one prescription written by a pediatric rheumatologist, 57 (14%) had at least one prescription written by an internist rheumatologist, 27 (7%) had at least one JIA prescription from a non-rheumatology specialist but none from a rheumatologist, and 41 children (10%) had prescriptions only from primary care providers. There was no significant difference between groups in the distribution of races. Conversely, the population of children in the pediatric rheumatologist group was significantly younger than that in the internist rheumatologist group (p < .001) (Table 1).

**Figure 2:**
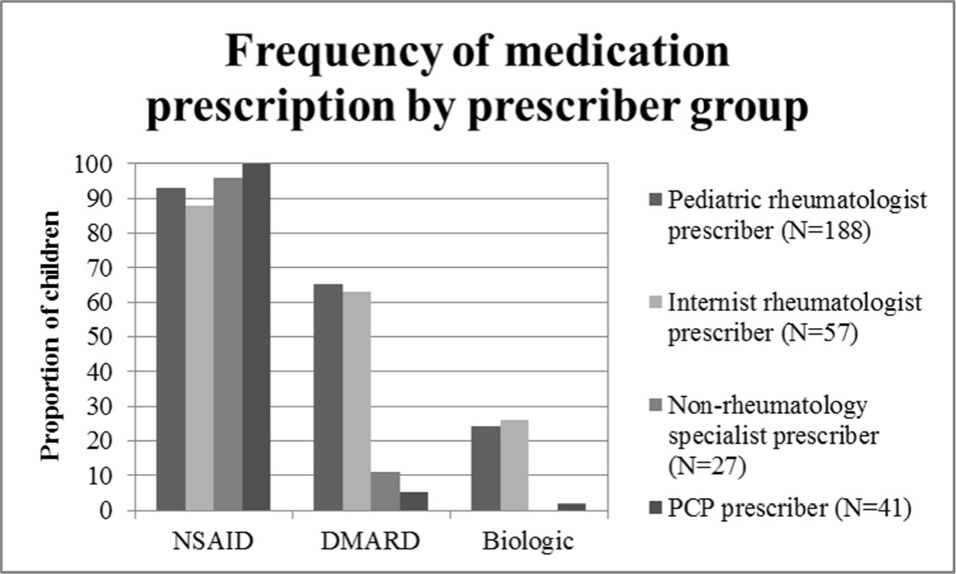
**Frequency of claims for JIA medication classes for children in each of the prescriber groups.** Children in the internist rheumatologist and pediatric rheumatologist groups were more likely to have claims for DMARDs (p < .001) or biologic agents (p < .001). There was no significant difference in the frequency of claims for any medication class when children in the internist and pediatric rheumatologist groups were compared.

While 94% of the children in the cohort had a claim for an NSAID filed during the study period, only 45% had a claim for a DMARD and 17% had a claim for a biologic agent. There was no significant difference between internist and pediatric rheumatologist prescribers in the frequency of claims for NSAIDs, DMARDs, or biologic agents; however, primary care physicians and non-rheumatology specialists were significantly less likely than rheumatologists to prescribe DMARDs (p < .001) or biologic agents (p < .001) (Figure 2). Separate analyses comparing internist and pediatric rheumatologist prescribers within age groups revealed no significant differences. The sensitivity analysis including only children 15 years of age or younger revealed the same pattern of findings.

**Table 1:** **Demographic characteristics of prescriber groups**

	Pediatric rheumatologist prescriber (N = 188)	Internist rheumatologist prescriber (N = 57)	Non-rheumatology specialist prescriber (N = 27)	PCP prescriber (N = 41)
Age				
0-5 years	27 (14%)	3 (5%)	0 (0%)	6 (15%)
6-10 years	43 (23%)	10 (18%)	2 (7%)	4 (10%)
11-15 years	76 (40%)	11 (19%)	7 (26%)	19 (46%)
16-21 years	42 (22%)	33 (58%)	18 (67%)	12 (29%)
Race				
Caucasian	109 (58%)	43 (75%)	15 (56%)	28 (68%)
African American	66 (35%)	8 (14%)	10 (37%)	10 (24%)
Other/unknown	13 (7%)	6 (11%)	2 (7%)	3 (7%)

## Discussion

In this study of Medicaid-enrolled children with JIA, the prescribing patterns of internist rheumatologists did not differ significantly from those of pediatric rheumatologists, despite the differences in their patient populations. The internist rheumatologist group included a higher proportion of older children, consistent with prior studies [4–6]. It is likely that internist rheumatologists are “self-selecting” a patient population similar to the young adults already in their practice; in contrast, internist rheumatologists may be hesitant to take on the challenge of younger patients, who may require different approaches to the physical exam and different dosing regimens for medications.

The finding that the prescribing patterns of internist rheumatologists were not significantly different from those of pediatric rheumatologists, is contrary to the direction of previous studies of pediatric versus adult primary care providers [11, 12]. The shared specialty-specific knowledge regarding rheumatology medications may ameliorate differences in prescribing patterns between internist and pediatric rheumatologists. It is also plausible that differences in prescribing patterns exist between internist and pediatric rheumatologists, but that they pertain to choice of specific agents and dosing regimens, rather than frequency of use for medication types.

Primary care providers and non-rheumatology specialists exclusively provided prescriptions for 17% of the cohort, and these children were less likely to be prescribed DMARDs and biologic agents. It would be appropriate for primary care providers and non-rheumatology specialists to limit their care to children who did not require more aggressive medications; however, as a claims analysis, this study did not have sufficient clinical information to determine whether this was the case.

This study is subject to several limitations. Although the study yielded a disease prevalence of 50 JIA cases per 100,000 children, consistent with previous studies [8], the overall number of children in each prescriber group was relatively small and limited the study’s statistical power. In addition, although steps were taken to minimize the risk of incorrect diagnosis, this study is subject to the possibility of miscoding. Prescriptions outside the study period and telephone consultations were not included, and thus we could have underestimated the involvement of specialized prescribers. We assumed that the most specialized prescriber would be directing a patient’s care, but that may not be accurate in all cases. Finally, these data are from 7/1/2005-6/30/2007, and it is possible that prescribing patterns may have changed since that time.

## Conclusions

Despite the limitations, these findings have implications for physicians who care for children with JIA. Although the patient populations cared for by pediatric and internist rheumatologists were different, the prescribing patterns for the two groups of providers were similar. Non-rheumatology specialists and primary care providers, on the other hand, were less likely to prescribe DMARDs or biologic agents. Given that early treatment with these agents has been associated with an improved likelihood of response [9, 10], primary care providers and non-rheumatology specialists should consider early referral to, or consultation with, a rheumatologist to ensure that their pediatric patients with JIA are receiving appropriate medications.

## Abbreviations

DMARDs: Disease-modifying anti-rheumatic drugs
HPSAs: Health professional shortage areas
JIA: Juvenile idiopathic arthritis
MUAs: Medically underserved areas
NSAIDs: Non-steroidal anti-inflammatory drugs.
